# Human-in-the-Loop Myoelectric Pattern Recognition Control of an Arm-Support Robot to Improve Reaching in Stroke Survivors

**DOI:** 10.1109/TNSRE.2025.3549376

**Published:** 2025-03-18

**Authors:** Joseph V. Kopke, Michael D. Ellis, Levi J. Hargrove

**Affiliations:** Department of Biomedical Engineering, Northwestern University, Chicago, IL 60611 USA. He is now with the Veterans Affairs Office of Research and Development, JBVAMC, Chicago, IL 60612 USA; Department of Physical Therapy and Human Movement Sciences and the Department of Physical Medicine and Rehabilitation, Northwestern University Feinberg School of Medicine, Chicago, IL 60611 USA; Center for Bionic Medicine, Shirley Ryan AbilityLab, Chicago, IL 60611 USA; Department of Biomedical Engineering and the Department of Physical Medicine and Rehabilitation, Northwestern University, Chicago, IL 60611 USA

**Keywords:** Rehabilitation robotics, human-in-theloop, stroke rehabilitation, myoelectric control, pattern recognition

## Abstract

The objective of this study was to assess the feasibility and efficacy of using real-time human-in-the-loop pattern recognition-based myoelectric control to control vertical support force or vertical position to improve reach in individuals with chronic stroke. This work attempts to move proven lab-based static arm support paradigms towards a controllable wearable device. A machine learning (linear discriminant analysis)-based myoelectric pattern recognition system based on movement intent as determined by real-time muscle activation was used to control incremental changes in either vertical position or vertical support force during a reach and retrieve task, with the goal of improving reaching function. Performance under real-time control of both options was compared to two unchanging static-support conditions (current gold standard) and a no-support condition. Both real-time control paradigms were successfully implemented and resulted in greater forward-reaching performance as demonstrated by increased elbow extension and horizontal shoulder adduction compared to no-support and was not different from the current gold standard static support paradigms. Muscle activation levels with real-time support were lower than the no-support condition and similar to those observed during the static support paradigms. Real-time detection of user intent was successful in controlling both vertical position and vertical support force and enabled greater reaching distance than without it demonstrating both its feasibility and efficacy albeit with some limitations.

## I. Introduction

WORLDWIDE, 12 million strokes occur each year, close to 90 million people have survived a stroke, and 36–44% of these survivors are left with chronic disability [1], [2], [3]. Most commonly, this entails upper extremity weakness, hypertonicity, loss of joint coordination, spasticity, and abnormal muscle timing [4], [5], [6], [7], each of which limits function and accomplishment of activities of daily living. While therapy and rehabilitation have resulted in small yet statistically significant improvement in performance, many survivors are left with chronic upper-extremity impairments leaving them wanting of a better solution.

Exoskeletal devices have been implemented to address the above impairments of the distal joints of the upper extremity, either to assist in therapeutic intervention or in accomplishing activities of daily living [8], [9], [10], [11], many implementing electromyographic (EMG) control [12], [13]. However, they have yet to be widely adopted by individuals after stroke. One reason may be that the severity of some of these impairments present in direct proportion to the amount of effort (neural signal sent to activate muscles) [14], [15], [16]. The harder one tries, especially when lifting their arm against gravity, the greater the impairment (e.g., reduced reach distance; increased co-activation, and increased unintentional flexion throughout the elbow, wrist, and hand) that these devices must then try to overpower, limiting their effectiveness.

Arm support (gravity compensation) alleviates the severity of these stroke-related impairments by reducing hypertonicity, abnormal synergy, and abnormal excitation, thus enabling more joint excursion, better coordination, and greater reaching [14], [17]. Thus, arm support is crucial in this population not only due to shoulder weakness but also to the negative functional effects caused by the significant effort required to lift and move the arm against gravity.

Research groups have used a broad range of *lab-based* devices, from simple surfaces or limb-weight support systems to complex robotic systems to support the arm against gravity in different ways, both to achieve and to explore this phenomenon [18], [19]. These efforts have led to the creation of tabletop and wheelchair *home-based* systems. Vertical support has generally been controlled by the research team and provided in an unchanging static manner. Thus, validating the feasibility and efficacy of human-in-the-loop (real-time or online) control of robotic support of the shoulder *after stroke* is an important next step in the process to move towards an independent wearable system – capitalizing on unweighting the arm yet not be limited by the lack of portability of current systems.

In one of the first efforts to study such a control system for the shoulder after stroke, Makowski et al. used a neural network to predict and apply *horizontal* planar forces during reaching on a modified HapticMaster with some success (i.e., slightly improved reach but impaired control and movement characteristics) [20]. They also reported an equal improvement in reach distance but no detriment to movement characteristics when a static *vertical* force offset, equal to only half of the participants’ limb weight, was applied. The decision to use horizontal forces contributed to negative performance characteristics while vertical support did not.

Weight compensation has since been explored through an EMG based adaptation control scheme [21], direct and indirect force control [22], and the use of anthropometric and model based parameter estimation [23] with varying success. Just et al implemented a control paradigm based on anthropometric data combined with force and torque information from a specific pose to maximize weight compensation with three participants with stroke and demonstrated improvement in reachable horizontal workspace and significant reduction in EMG. These results, in addition to the above studies [14], [17] led us to explore real-time myoelectric control of *vertical* support.

The use of EMG as a control signal may be advantageous for this application. Using participant muscle signals ensures active use and participation eliminating slacking and disuse, a problem for many rehabilitation and assistive devices [24], [25]. Additionally, if implemented properly it could avoid the negative effects of exerting too much effort – leading to unintentional contractions and may even help the user identify and learn to avoid these abnormal synergies. Implementing the *independent* use of myoelectric control away from the laboratory may require the use of implantable electrodes, specifically for unreachable muscle sites.

Here we demonstrate real-time myoelectric pattern recognition control of a robotic arm (modified HapticMaster) to provide two types of vertical support in a small sample (n = 5) of individuals with chronic moderate stroke. The goal of this study was to test the feasibility of this approach and enable a preliminary analysis of its effectiveness in improving reaching ability and reducing shoulder muscular effort. We hypothesized that real-time control of vertical support, through either position or force, would enable greater elbow and horizontal shoulder adduction excursion compared to the no-support condition, as well as reduce total shoulder muscle activity. The tested real-time support conditions (simulating what might be expected of or necessary for a usable wearable device) were also compared to the current gold-standard lab-based interventions of frictionless rigid horizontal plane and force-based full limb-weight support – these types of support were expected to demonstrate the maximal reach possible for each participant with this type of intervention. This work expands on the ideas presented in conference form [26], specifically with an increased number of participants, more expansive analysis, and greater detail in methods.

## II. Methods

### A. Participants

Five participants consented, enrolled, and completed this study, which was approved by the Northwestern University IRB (IRB# STU00210805). Inclusion criteria included having had a unilateral stroke more than six months before the study, with motor deficits limited to one side, and having moderate motor impairment as determined by the upper-extremity portion of the Fugl-Myer Assessment (10*<*score*<*40). Participants also had to be able to follow a set of 4-step sequential instructions. Exclusion criteria included lack of any shoulder or elbow volitional control or proprioception, any range of motion limitations that prevented safe participation and interaction with the parameters set for the robot (100° of shoulder abduction/scaption with neutral humeral rotation), any shoulder or spine joint pain, and any major medical conditions that may preclude safe participation.

### B. Experimental Setup

**Fig. 1.**
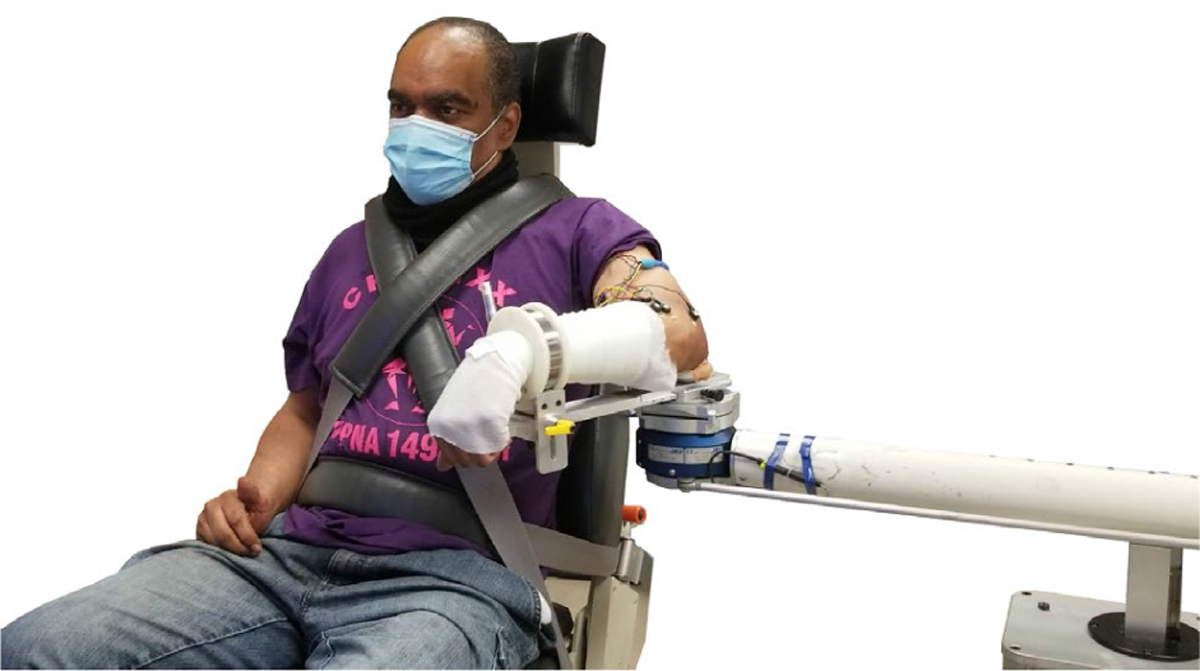
Setup. Participant setup in the ACT^3D^. Arm resting on lower floor at 70° abduction.

The participant was positioned in the Biodex chair (Biodex Medical Systems, Inc., Shirley, NY), with their affected arm/shoulder abducted 90° and horizontally adducted 45° from the frontal plane. The participant was then strapped into the Biodex chair with nylon belts to constrain movement of the upper body and torso. A lightweight fiberglass cast was applied to the participant’s paretic forearm (not crossing the elbow or wrist joints) and then attached to the ACT^3D^ haptic device end effector via custom hardware (Fig. 1). The ACT^3D^ consists of the admittance-controlled HapticMaster robot (Moog Inc., The Netherlands) with a six-degree-of-freedom load cell end effector (JR3, Woodland, CA).

The robot was programmed to allow free unconstrained movement in the transverse (horizontal) plane (including horizontal shoulder abduction/adduction and elbow flexion/extension). Internal and external rotation were constrained due to the design of the gimbal and the way the participant was secured to the robot. Virtual rigid surfaces were programmed to limit the amount of vertical movement to between 70° and 100° shoulder abduction to protect the shoulder.

Twelve pairs of Ag/Ag-Cl gel EMG electrodes were placed over the following muscle sites, according to the guidelines in the *Anatomical Guide for the Electromyographer*: anterior, middle, and posterior deltoid, upper-trapezius, pectoralis major, supraspinatus, infraspinatus, and biceps brachii, with two pairs over the wrist and finger extensors and two over the flexors. A custom amplifier based on the Texas Instruments ADS1299 was used to sample EMG at a frequency of 1 kHz, with a gain of 1k, and bandpass filtering of 20–50 and 70–350Hz.

Within this setup each participant completed a lift and reach task (section C) under five different support conditions (section D), two of which used real-time control (sections E, F, G). Three of five participants completed an offline unrelated movement task (section I).

### C. Lift and Reach Task Description

Within this setup ten lift-reach-return-lower trials were performed under each of five conditions. The participant was required to lift their arm to achieve a vertical target window between 80 and 100 degrees of abduction, reach out straight in front of them as far as they could, return to the starting position, and lower their arm below 80° abduction. Although ultimately arbitrary, these limits were implemented to protect the glenohumeral joint and because many compensations can accommodate inaccuracies in shoulder control. Verbal instructions to participants generally included phrases such as “lift, reach out in front of you as far as you can, return, and lower your arm.” No mention was made of speed, as ballistic motions are not used functionally. Each participant completed all five support conditions in a randomized order. Data recorded for each trial included calculated joint angles, position and velocity of the end effector, the forces and moments at the load cell under the end effector, and 12 channels of surface EMG.

### D. Support Conditions

The lift and reach task was completed under the following five conditions: a no-support condition, a tabletop condition, a full limb-weight supported condition, a real-time vertical velocity (Δ position) control condition, and a real-time force control condition.

The no-support condition simulates typical ability without assistance requiring the participants to lift the full weight of their limb during the entire task. The tabletop and full limb-weight support were included as a gold-standard and theoretical maximum as they have been shown to offer the greatest improvement in reach distance – hypothetical best cases for comparison with the real-time control data. The tabletop is a frictionless horizontal haptic surface created by the ACT^3D^ that the participant rests their arm on within the target window. The full limb-weight support condition required the ACT^3D^ to supply an upward vertical force equivalent to the weight of the limb.

The two real-time controllers implemented myoelectric pattern recognition to induce a change in the robot vertical position or the robot supplied vertical force every 25ms. These are two possible control schemes for a future powered wearable device.

### E. Position- and Force- Controller Description

A linear discriminant analysis–based pattern recognition controller consisting of three classes (abduction, adduction, and no movement) was used to control the robot in the vertical direction in two different ways: position control and force control. The position-based controller consisted of applying classifier output (class and speed) to the vertical position of the robot at each decision window (25ms) using the previous 200ms of data. More explicitly, a velocity was applied across each 25ms window that was proportional to EMG activity (over the previous 200ms) as described in Equations 1, 2, and 3 from Scheme et al. [27]. Similarly, for the force-based controller, incremental changes were made to the vertical force using the class and magnitude output from the proportional control classifier. In this way, the robot responded to the predicted intent (over the previous 200ms) of the user each 25ms.

(1) $$PC_{i}={\left(\frac{1}{C_{i}}\sum_{j=1}^{N_{CH}}\;S_{i,j}MAV_{j}\right)}^{2}$$

where $PC_{i}$ is the proportional control output for each class for a given window, $N_{CH}$ is the number of channels used, $MAV_{j}$ is the mean absolute value of channel j within the given window, $S_{i,j}$ is the stored set of values representing the *centers* of each class and channel that were calculated and stored during classifier training by (2)

(2) $$S_{i,j}=\frac{1}{K_{i,j}}\sum_{k=1}^{K_{i,j}}MAV_{i,j,k}^{Tr}$$

where $MAV_{i,j,k}^{Tr}$ is the mean absolute value of the training data from class i, channel j, and computation window k, and $K_{i,j}$ is the total number of computation windows (k) for class i and channel j. Ci is the stored set of per-class *normalization factors* calculated and stored during classifier training by using (3). These are the channel-sum of squared *centers* found using (2) in the following:

(3) $$C_{i}=\sum_{j=1}^{N_{CH}}S_{i,j}^{2}$$

**Fig. 2.**
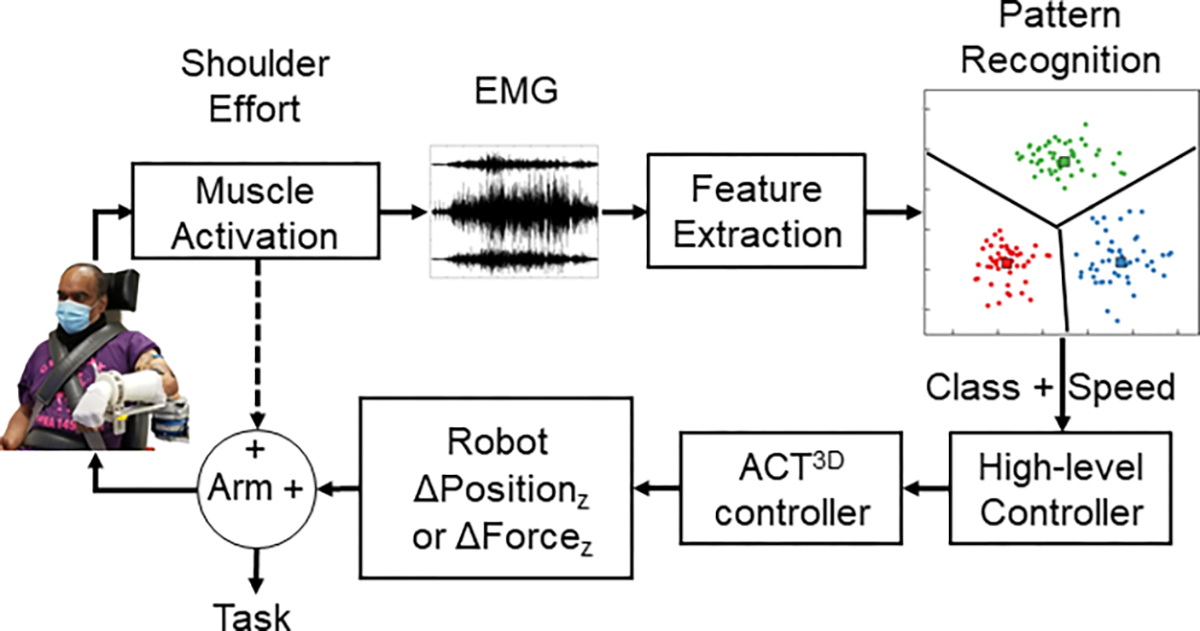
Block diagram of control schemes. Dashed line indicates that only during the force-based control can muscle activity *directly* affect the movement or stiffness of the arm.

Additional gains to the proportional control for each class and controller type were adjusted to each participant based on the following guidelines: gains were lowered if the participant consistently lifted their arm directly into the ceiling limit upon first effort to lift their arm; conversely, gains were increased if their elbow was lifting off the load cell or it appeared that the participant was waiting on the robot to respond. Different gains were used for the position and force paradigms as well as between the positive and negative vertical directions. A graphical depiction of the control scheme is presented in Fig. 2.

### F. Control System Training

Training data consisted of three sets of two, 10-second trials of each movement type (abduction, adduction, no movement) with each set occurring at a different horizontal adduction position (0°, 45°, and 90°*)*. Additionally, five supported reach trials on a rigid table-like surface at 90° abduction and five limb-weight supported reach trials were included in the no-movement class training data for the position and force controllers respectively. Supported reaching trials were included to aid the classifier in discriminating between pure abduction and adduction and reaching, since common muscle activation patterns across movements, as well as generalized increased muscle tone during tasks occur after stroke. In total, 28, 10-second trials were used as training data.

### G. Data Segmentation and Feature Extraction

The same procedures were used for segmentation and feature extraction for both online and offline classifiers and analyses. All 12 channels of EMG were processed using 200ms windows and 25ms steps (175ms overlap). Within each window, the Hudgin’s feature set (mean absolute value, number of slope sign changes, number of zero-crossings, and waveform length) was extracted in addition to 6^th^-order autoregressive features. In total, 12 channels, each with 10 feature vectors, for a total of 120 features, were used to train and test both the online and offline classifiers [28]. The minimum Mahalanobis distance between the current window compared to the 3-class training data determined the class output.

### H. Reaching Task Data and Statistical Analysis

**Fig. 3.**
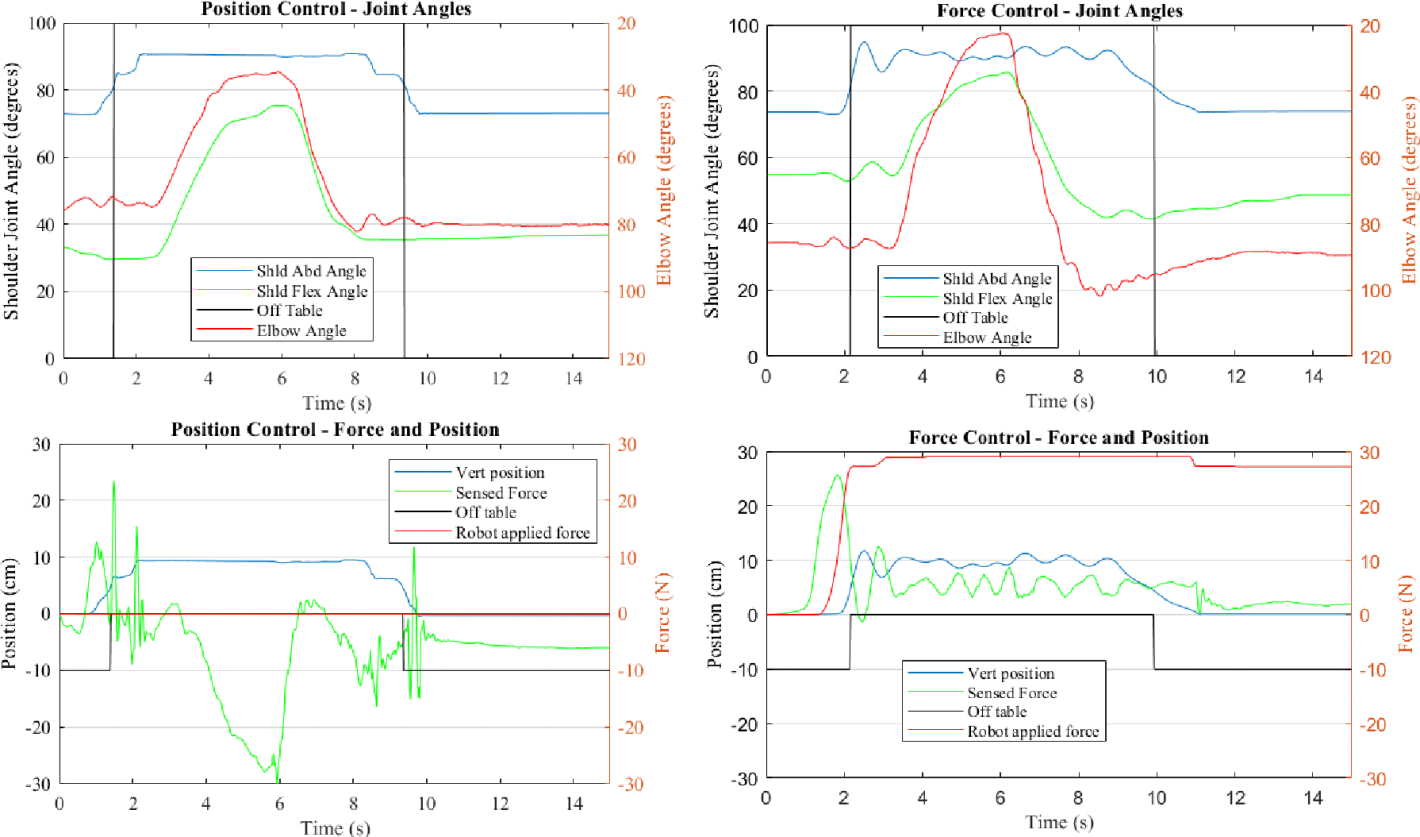
Representative trials. Representative lift and reach trials of each control type: position control (left) and force control (right) with joint kinematics (top) and robot forces (bottom). The data in red uses the right axis, while all other data use the left axis. The large negative sensed force (green) displayed in the bottom left is a participant engaging an abnormal synergy pattern in order to maximize reach; the controller successfully identified the reach and prevented the robot from lowering its vertical position outside of the target window to enable a greater reach. In the bottom right at the end of the reach around 11 seconds can be seen a minimal drop in support force (red) as the classifier had difficulty recognizing adduction since the arm only needs be relaxed to lower the arm to the start position when the support is less than the weight of the limb.

Figure 3. depicts kinetic and kinematic data for a typical trial using both the position and force controller. Reaching task data was analyzed for both elbow joint extension and shoulder joint horizontal adduction in all 10 trials of all five conditions. Primary outcome measures were the maximal joint range of motion attained during each trial while the arm was within the target vertical window. Subject means and standard deviations are listed. T-tests were completed for the a priori primary comparisons between each real-time controller and the no-support condition for both the elbow and shoulder joints. Comparisons were also made to determine if a difference could be detected between each controller type and its gold-standard counterpart. One-sided tests were used because it was expected that no-support performance would be worse than supported and that the gold standard static support would perform the best.

EMG signal amplitude was also recorded to assess relative effort between conditions. EMG data were rectified, and a moving average was applied using 200ms windows, followed by normalization of each channel to the maximal value recorded throughout each study session. Normalized EMG from all trials of each condition and participant were averaged. A time series heat map depicting relative EMG activity for four channels involved in lifting the arm against gravity (anterior, intermediate, and posterior deltoid and upper trapezius) across all trials of each condition was created. These data indicate the amount of proximal muscle activation required during the reaching task for each condition and enable a qualitative analysis of muscle effort.

To gain insight into the total shoulder effort or muscle activity required to perform the tasks under each support condition, the normalized EMG data were summed across trials and all four shoulder muscles (anterior, intermediate, posterior deltoids, and supraspinatus). Two t-tests were then completed to compare each of the online support conditions to the no-support condition. Two additional t-tests were completed to compare the gold standard counterparts with the controllers.

### I. Unrelated Movement Task

As well as providing intended support, the controller should avoid directing unintended changes in support during other unrelated movements such as movement at the elbow, hand, or shoulder like bringing the arm across the chest (horizontal adduction). As the increased tone and common muscle activity patterns that emerge after stroke might prevent successful discrimination between shoulder abduction or adduction (vertical movements) and other movements, we also explored the feasibility of using pattern recognition to control a wearable exoskeleton while the upper extremity is moving in other directions.

Additional data acquired from a subset of study participants (n = 3) were examined to determine the ability of the classifier to identify untrained and unrelated (i.e., not vertical abduction or adduction) activities as “no-movement”. Four combinations of tasks were selected and accomplished in random order: horizontal shoulder adduction and abduction, internal and external (isometric) rotation, elbow flexion and extension, and hand open and close. Each combination of tasks was accomplished in an alternating fashion under two different conditions, limb-weight support, and tabletop support, simulating the ideal support that could be provided via the real-time controllers. Internal and external rotation were isometric so participants were asked to “use about the same strength that is required to move your arm like this” (and an external rotation motion moving from neutral to 90° while the humerus was abducted to 90° was demonstrated). As above, limb-weight support entailed the robot providing a supportive force equivalent to 100% of the limb weight while the tabletop support was a rigid and frictionless horizontal haptic plane, similar to a smooth table.

Forty-five seconds of each combination of tasks was recorded during three, 15-second trials. EMG data were subsequently classified offline using the classifier established for the real-time control described above. Classification of these motions results in one of three possible classes (abduction, adduction, or neither/no-movement). Since this control scheme is concerned with only controlling vertical support, ideally all data from these other movements would classify as no-movement. Successful discrimination would indicate that the characteristics of these tasks are more similar to the no-movement class than to the abduction and adduction movement classes and would eliminate the need to include training data from these motions into a future classifier.

Poor performance would indicate the need for additional movements to be included in the training for the no-movement class.

## III. Results

### A. Range of Motion

To evaluate the usability and efficacy of myoelectric control of arm-weight support after stroke, we implemented an EMG based pattern recognition controller operating in two different modes – changing either the position or the support force in a lift, reach, and retrieve task. Figure 3 depicts joint kinematic data as well as forces provided and sensed during a position control trial as well as a force control trial. As seen in the top left and top right of Fig 3. shoulder joint angles use the left y-axis while the elbow uses the right. The vertical black lines indicate when the arm is within the target window of 80 to 100° of shoulder abduction. Once the arm is in the vertical target window the participant reaches forward as indicated with the changes in horizontal shoulder flexion and elbow extension angle. The position controller holds the arm rigid unless it detects an EMG pattern directing a change hence there is much less variation in position throughout the trial compared to the force control on the right.

Robot vertical position as well as sensed and applied forces are plotted in the bottom left and bottom right of Fig 3. The sensed force at the JR3 sensor is noisy for the position control (bottom left) as the position is incrementally changed each 25ms when the abduction or adduction EMG pattern is detected. The position control enabled this participant to engage his or her extension synergy. As the participant reached forward you can see the sensed force (green) decreasing to approximately −30N indicating they were pressing their arm down into the rigid surface. Once they started to retract their arm the force immediately returned to close to zero. Again, we see the noisy sensed force data as the position was lowered back down out of the target window completing the task. In the force controller condition, we see a bit of oscillation in the sensed force and vertical position as the participant attempted to keep their unweighted arm (supported by close to 30N) in the target window during the reach task. Since the robot was supporting the weight of the arm, minimal force is required to lower it as gravity assists. The controller was unable to detect the participant’s desired intent to lower their arm – robot applied force remains close to 25–30N throughout the trial (seen in red) while the participant was still successful in lowering their arm.

**Fig. 4.**
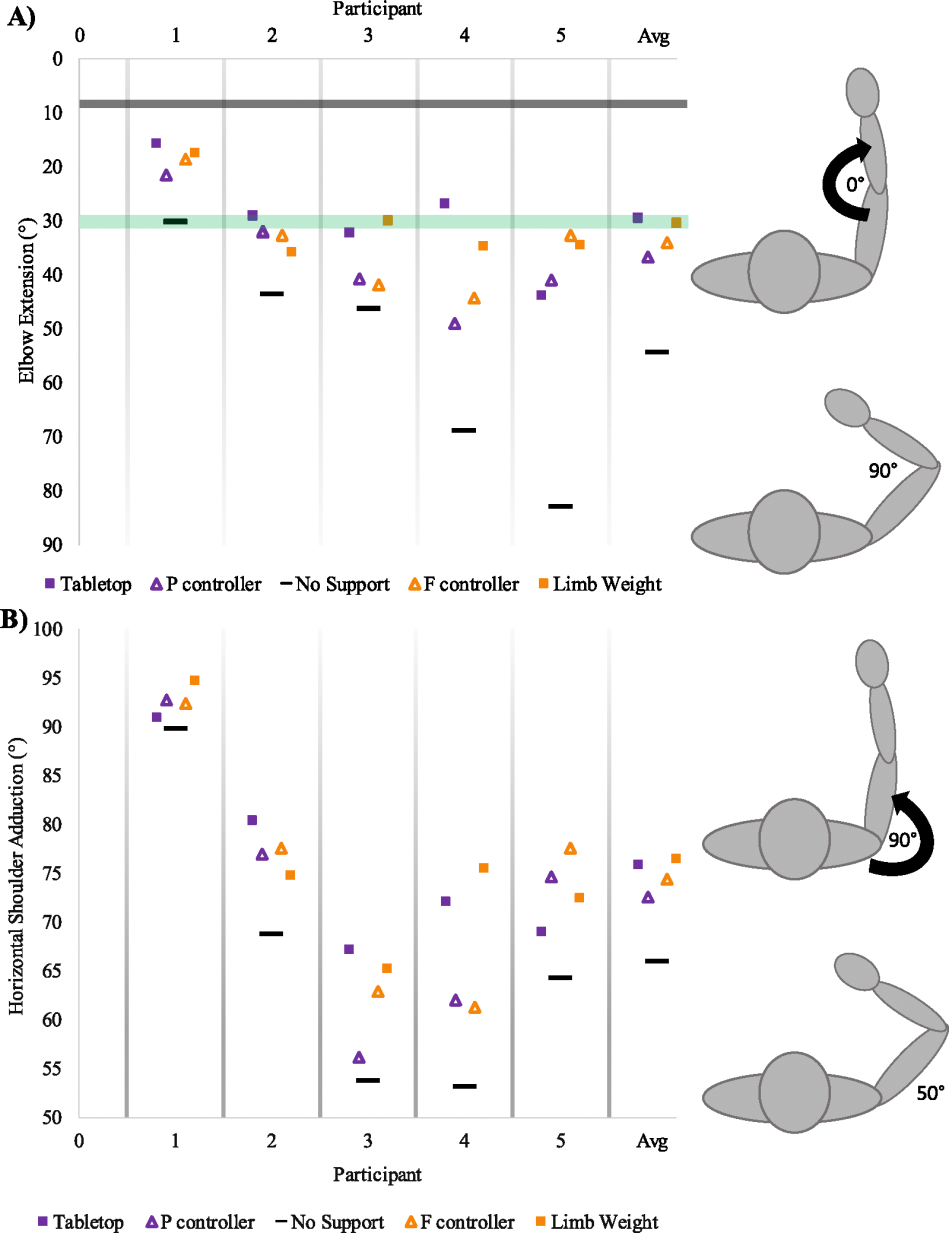
Maximum Reach Characteristics. A) Maximum elbow extension and B) maximum horizontal shoulder adduction attained during forward reach under five conditions: Tabletop support (arm resting on rigid frictionless horizontal surface provided by robot), ΔP controller (position based real-time control), No-support (participant lifting the full weight of limb), ΔF controller (force based real-time control), Full Limb-Weight Support (by the robot). Averages of each condition are provided on right side. Squares represent static (unchanging) support conditions, while triangles represent dynamic (changing) support conditions based on the output of the controller. Purple represents position based support and orange represents force-based support. The green horizontal bar indicates functional elbow extension [35], and grey bar indicates near-full elbow extension.

To evaluate efficacy we compared shoulder and elbow joint excursion during the task under five different conditions. Figure 4. displays the maximal joint angles during the five support conditions as well as the average performance within each support condition. Figure 4a displays the joint excursion for the elbow, moving from a flexed position to their attempted full extension and returning to a flexed position. Figure 4b displays horizontal shoulder adduction starting from a position close to 30° (slightly forward of neutral or 0° horizontal shoulder flexion) and increasing towards 90° as the participants reach in front.

Tables I and II show the joint angle mean and standard deviation (degrees) for the reach and retrieve task for each participant during the five support conditions for elbow excursion and shoulder excursion respectively. Generally, the standard deviation was within error tolerances of joint angle measurement and values between conditions were close indicating consistent repeatability under all support conditions.

To evaluate the change in *elbow joint excursion* a one-sided paired t-test indicated there is a significant large difference between the position and no-support conditions (position: 41.5(10.4) and no support: 59.1(21.9), p = 0.030) and a significant large difference between the force control and no-support condition (force: 37.7(10.4) and no support (59.1(21.9), p = 0.037).

To evaluate the difference in shoulder joint excursion a one-sided paired t-test indicated there is a significant large difference between the position and no-support conditions (position: 67.3(14.8) and no support: 61.8(14.9), p = 0.032) and a significant large difference between the force control and the no-support conditions (force: 69.7(14.1) and no support: 61.8(14.9), p = 0.013).

Paired t-test comparisons were also made with the hypothetical gold-standard static conditions of tabletop support and unchanging full-limb weight support compared to their online controller counterpart. A significant large difference was found between table support and real-time position control for elbow excursion (table: 34.9(10.6) and position 41.5(10.4), p = 0.04) but not for the shoulder joint nor either joint comparison with the force controller.

### B. Muscle Activation

**Fig. 5.**
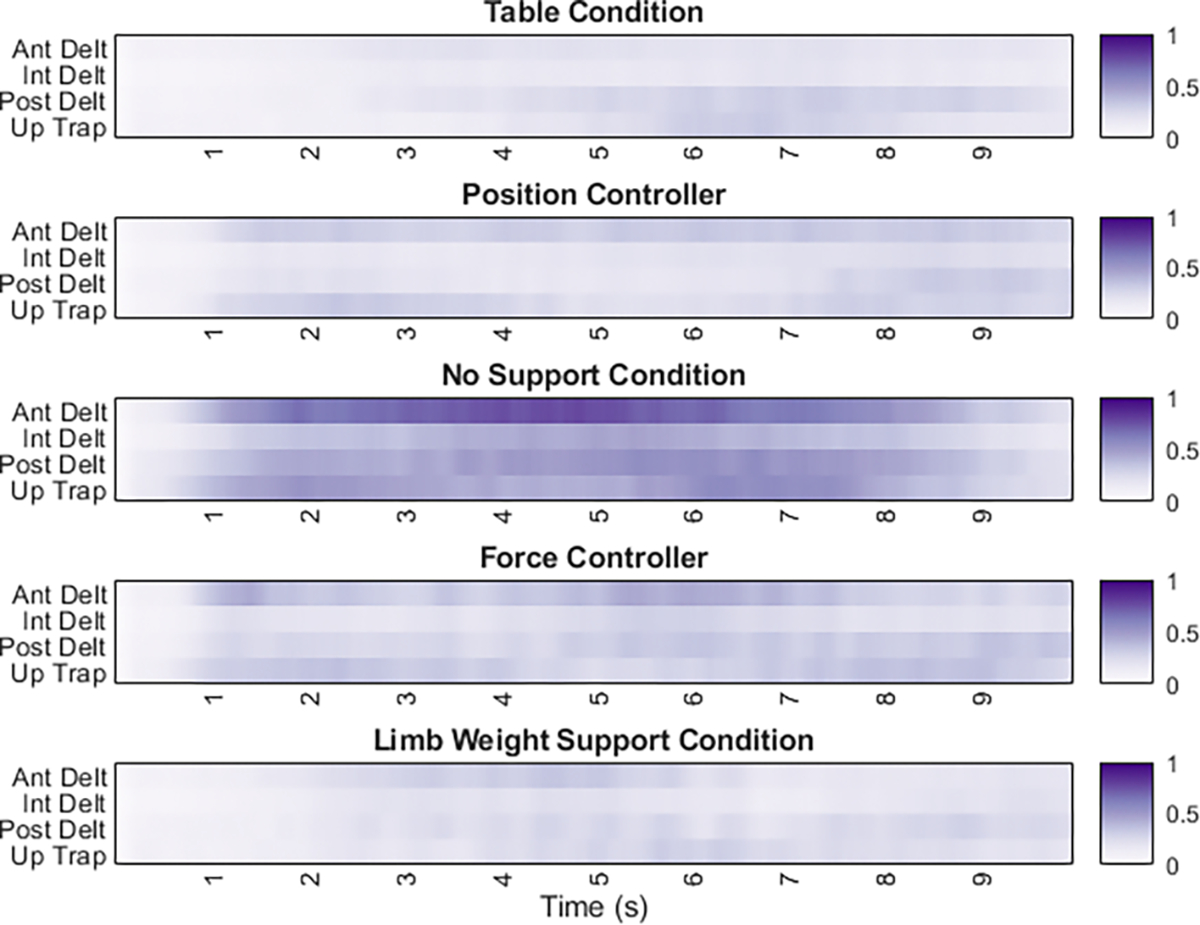
Average normalized EMG during each condition Averaged smoothed (200ms) normalized EMG is shown for all participants across all trials. Ant – Anterior, Int – Intermediate, Post – Posterior, Up – Upper, Trap – Trapezius. Note that the no-support condition is positioned in the middle to allow easier comparison between the support paradigms.

To evaluate the amount of shoulder muscle activation during each support condition, we recorded EMG from the three heads of the deltoid and the upper trapezius. Figure 5. displays an EMG heatmap for the reach and retrieve task to qualitatively show differences in muscle activity between the support conditions. The darker the shade, the greater the muscle activity. The gold standard conditions have a small yet detectable reduction in muscle activity compared to the online controller conditions, which both have marked reductions in activity compared to the no-support condition.

**Fig. 6.**
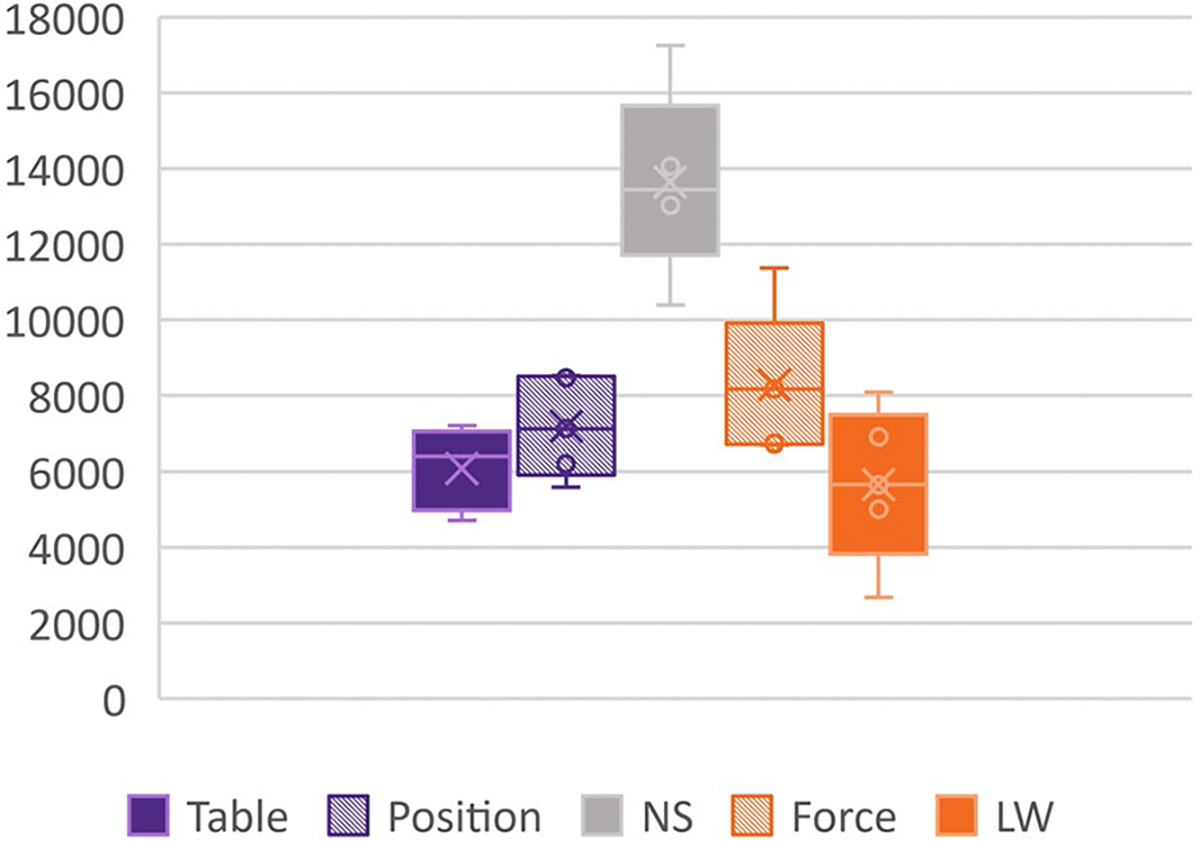
Cumulative normalized shoulder muscle activity. Box plot of summation of four channels of shoulder EMG data (3 heads of the deltoid and the supraspinatus) normalized to their respective participant maximums summed across all trials for each support type. NS – no-support, LW – limb weight support.

Figure 6. depicts a box plot of the cumulative normalized shoulder muscle EMG for all trials under each support condition. This includes the activity of the three deltoids and the upper trapezius. While the gold standard static support paradigms had the least EMG, the online controllers averaged a little more than 50% of the shoulder muscle activity compared to the no-support condition.

To evaluate the change in EMG a one-sided paired-t test indicated that there is a significant large difference between the summed total shoulder EMG for real-time position control and the no-support condition (position: 7188.8(1315.6) and no support: 13633.6(2458), p = 0.001), and between force control and no-support (force: 8287.9(1905.3) and no support: 13633.6(2458), p *<*0.001). EMG comparisons are not included between the static and controlled conditions because the controller required EMG for input while the static support conditions did not require the use of shoulder muscles to lift.

### C. Feasibility

To evaluate the feasibility of our classifier in identifying user intent of motions to be left unassisted or ignored, we tested the classifier on a sampling of untrained tasks while the arm was fully supported. Classification was done offline to inform future online applications. Tables III (device providing limb-weight support) and IV (device providing tabletop support) show the classification percentage of all data windows for each unrelated movement that was classified as one of these three movement class options. Significant error is present under both controller paradigms. To evaluate the *potential* of offline classification (best case scenario) using this experimental setup and data we averaged the best performance of each participant for each task. This resulted in classification accuracies of 91, 90, 100, and 94%, respectively, for the four tasks during the limb-weight support condition and 84, 89, 96, and 85%, respectively, for the four tasks during the tabletop support condition.

## IV. Discussion

In this study, we assessed the feasibility of using human-in-the-loop myoelectric pattern recognition controllers to control a robot that provided vertical shoulder support after stroke, to enable the user to accomplish a voluntary forward-reaching task. The controllers were used to provide vertical support via position or force, both known to improve reaching performance when provided statically [29]. Additionally, we examined EMG activity under each condition and, in a subset of participants, the ability of the classifier to discriminate other non-related tasks.

Reaching performance, both elbow excursion and shoulder horizontal adduction excursion improved in all participants for both controllers compared to the no-support condition. This indicates that each participant was able to interact with the pattern recognition controllers well enough to accomplish the task and that both types of support significantly improved reaching performance compared to no-support for both elbow and shoulder excursion. A difference was also detected between the static table support and the online position controller, indicating online control is not as effective as a static table. This makes sense because with static control participants can engage an extensor synergy by adducting against the surface causing or enabling greater elbow extension. The online controller would not allow this, as the classifier would observe the adduction and initiate a lowering of the arm. This would be optimal therapeutically as it could help survivors of stroke to avoid engaging this abnormal synergy.

Of note, while the static conditions are considered the gold standard for lab-based applications, they are impractical for implementation in a wearable device. The tabletop condition would cause the arm to constantly be maintained at 90° of abduction (impractical) and the full limb-weight support condition would support the arm at 100% of its weight at all times either using up all battery power, interfering with daily activities, or at the very least annoying the user.

Participants 3 and 4 showed a marked reduction in reaching performance (Fig. 4a and 4b) when using the dynamic support offered by the controllers (triangles) compared to static support (squares). We postulate that this difference could be reduced through additional time using and getting accustomed to the control system [30], [31].

When participants used the online controllers, EMG activity was significantly reduced indicating that the participants were using less effort than during the no-support condition and close to that of the static support conditions. This manifested not only as less muscular effort but also as improved reaching performance. Furthermore, this indicates that the participants were working with, rather than against the robot during the specified tasks. These results are similar to those of Lenzi et al. who used proportional surface EMG control of robotic assistance at the elbow [32].

While there was a significant difference between table support and the position controller for elbow excursion, there was not for EMG between the position controller and table support. This could mean that the reduced reach for the controller was not due to shoulder effort but rather due to the utilization of the extensor synergy as mentioned above.

Our control paradigm represents an advancement in human in the loop control for this patient population in order to improve function with potential application to future wearable systems. Makowski et al. attempted to use EMG to control anterior-posterior horizontal reaching forces with robotic assistance [20]. They found vertical support was key to improving reaching ability but only applied vertical support up to half the weight of the limb. Our technique enables real-time control of vertical support force up to the entire weight of the limb which based on prior study results [14], [33] should enable greater improvement in reaching ability compared to supporting only half the weight of the limb. Other groups have examined the classification of shoulder movements but without application to real-time control of a device [34], [35], [36].

Qualitative feedback from participants on how they felt about using real-time control included the following comments: “this is great, I feel like I am hardly working at all,” “the first one [referring to the position-based controller] was a bit jerky, kind of like driving a big truck,” and “I really like this one [the force- based controller] since it is smoother and helps me reach further without having to work so hard.” Modifications to the control scheme, including the possible use of velocity filters or rate-limiters, may reduce the feeling of jerkiness but this would require further development and testing.

Using the classifier during unrelated (and untrained) motions resulted in high error rates in offline tests. The precise relationship between online and offline performance for pattern recognition myoelectric controllers is not well understood. In online control tasks, subjects have the opportunity to correct small mistakes, which may not affect the completion of the task but this has not been tested for individuals with stroke. The ability of participants with abnormal synergy to modify their muscle patterns or make adjustments in real time may be more limited. The average offline error rates were below what is necessary for a usable controller, but the *best-case* offline error rates for each participant were within the range expected for a usable online controller [37] and indicate what may be possible even without real-time adjustments – it may just take additional time to learn to be reproducible. The high error rates, especially between seemingly independent tasks (hand-open and close vs control of shoulder ab- and adduction), highlights the challenges of abnormal synergies experienced after stroke. Additional training data incorporating these or other unrelated movements to the classifier should improve the ability to discriminate between abduction, adduction, and other unrelated movements as was the case in this study for elbow flexion and extension. More work is needed to determine the ideal data set to maximize function and minimize error and training time.

Study limitations include the small number of participants, which limits the generalizability and statistical power of the study, and the absence of a shoulder tracking task, although we believe there are many ways in which the body can be positioned and moved to accommodate and compensate for minor errors in position. Comparison of the efficacies of the two types of control (force and position) is not possible without a larger cohort. Additionally, the offline analysis of the unrelated movements offers limited insight aside from the fact that initial results were mixed. An online analysis in which the user can learn and attempt to adjust to misclassifications would be a logical next step.

## V. Conclusion

Ultimately, we have shown that real-time control of vertical support via myoelectric pattern recognition is feasible and efficacious after stroke despite abnormal muscle activation patterns. Both the position and force-based controllers improved elbow and shoulder joint excursion during forward reach compared to the no-support condition, enabling greater reaching distance. Providing support reduced the effort needed to counter gravity throughout the task, reducing muscular activity by close to 50% and the subsequent movement impairments experienced post-stroke.

Challenges remain regarding full implementation, specifically how well a device could be controlled with free movement considering the significant error during untrained tasks. Immediate next steps include a multi-session study to determine the extent to which the use of the controller can be learned to maximize reaching and an online assessment of the interaction between the participant, the classifier, and unrelated movements. Including additional EMG channels and even force sensitive resistors followed by optimization may also be beneficial. Future steps include the design and application of these techniques to a wearable device that supports the shoulder in similar ways.

Related work could incorporate these techniques into a rehabilitation program helping users to identify and avoid abnormal muscle activation and movement patterns as they recover. Optimization of limb support could be systematically implemented thereby mimicking “progressive abduction loading therapy” proposed by Ellis et al. [33], [38] but utilizing a direct physiological signal as opposed to task performance to progress the intervention.

## Figures and Tables

**TABLE I:** Elbow Joint Excursion

	Participant				
Condition	1	2	3	4	5

Table	20 (4.7)	32 (2.0)	35 (2.7)	37 (7.7)	50 (4.4)
**Position**	**27 (5.1)**	**35 (2.0)**	**45 (1.5)**	**53 (3.2)**	**48 (4.5)**
No-support	35 (4.0)	49 (5.8)	49 (1.5)	72 (2.6)	91 (3.8)
**Force**	**23 (2.2)**	**36 (1.8)**	**45 (2.2)**	**50 (3.5)**	**36 (1.8)**
LW	20 (3.0)	37 (1.1)	35 (5.8)	40 (4.2)	45 (7.6)

**TABLE II:** Shoulder Adduction Joint Excursion

	Participant				
Condition	1	2	3	4	5

Table	88 (1.8)	73 (4.2)	62 (3.2)	65 (4.9)	66 (2.5)
**Position**	**87 (3.7)**	**74 (2.6)**	**52 (3.1)**	**54 (7.7)**	**70 (2.9)**
No-support	86 (2.4)	63 (4.4)	50 (2.5)	49 (2.7)	61 (2.3)
**Force**	**88 (2.5)**	**75 (1.5)**	**58 (3.3)**	**54 (5.4)**	**75 (1.5)**
LW	89 (4.0)	70 (3.8)	54 (7.8)	70 (3.9)	67 (3.4)

LW – Limb weight Support

**TABLE III:** **Limb-Weight Support** Classification of EMG Patterns During Unrelated Movements With Limb Weight Fully Supported. (%)

Classification of EMG patterns during unrelated movements with limb weight fully supported. (%)			
Unrelated Movement Classification	No Movement	Abduction-vertical	Adduction-vertical

Horizontal Add/Abd	78	6	16
Internal/External Rot	70	15	15
Elbow Flex/Extend	96	3	1
Hand Open/Close	84	16	1
Average – all tasks	82	10	8

**TABLE IV:** **Rigid Tabletop Support** Classification of EMG Patterns During Unrelated Movements With Arm Resting on Tabletop. (%)

Classification of EMG patterns during unrelated movements with arm resting on tabletop. (%)			
Unrelated Movement Classification	No Movement	Abduction-vertical	Adduction-vertical

Horizontal Add/Abd	63	13	23
Internal/External Rot	74	20	6
Elbow Flex/Extend	88	7	6
Hand Open/Close	75	24	0
Average – all tasks	75	16	9

Add - Adduction, Abd - Abduction, Rot - Rotation

## References

[1] Tsao CW , “Heart disease and stroke statistics–2023 update: A report from the American heart association,” Circulation, vol. 147, no. 8, pp. e93–e621, Feb. 2023, doi: 10.1161/cir.0000000000001123. 36695182 PMC12135016

[2] Hankey GJ, Jamrozik K, Broadhurst RJ, Forbes S, and Anderson CS, “Long-term disability after first-ever stroke and related prognostic factors in the Perth community stroke study, 1989–1990,” Stroke, vol. 33, no. 4, pp. 1034–1040, Apr. 2002, doi: 10.1161/01.str.0000012515.66889.24. 11935057

[3] Hardie K, Hankey GJ, Jamrozik K, Broadhurst RJ, and Anderson C, “Ten-year risk of first recurrent stroke and disability after first-ever stroke in the Perth community stroke study,” Stroke, vol. 35, no. 3, pp. 731–735, Mar. 2004, doi: 10.1161/01.str.0000116183.50167.d9. 14764929

[4] Kamper DG, Fischer HC, Cruz EG, and Rymer WZ, “Weakness is the primary contributor to finger impairment in chronic stroke,” Arch. Phys. Med. Rehabil, vol. 87, no. 9, pp. 1262–1269, Sep. 2006, doi: 10.1016/j.apmr.2006.05.013. 16935065

[5] Persson CU, Holmegaard L, Redfors P, Jern C, Blomstrand C, and Jood K, “Increased muscle tone and contracture late after ischemic stroke,” Brain Behav, vol. 10, no. 2, p. 01509, Feb. 2020, doi: 10.1002/brb3.1509. PMC7010575 31893564

[6] Dewald JPA, Pope PS, Given JD, Buchanan TS, and Rymer WZ, “Abnormal muscle coactivation patterns during isometric torque generation at the elbow and shoulder in hemiparetic subjects,” Brain, vol. 118, no. 2, pp. 495–510, 1995, doi: 10.1093/brain/118.2.495. 7735890

[7] McPherson JG, Ellis MD, Heckman CJ, and Dewald JPA, “Evidence for increased activation of persistent inward currents in individuals with chronic hemiparetic stroke,” J. Neurophysiol, vol. 100, no. 6, pp. 3236–3243, Dec. 2008, doi: 10.1152/jn.90563.2008. 18829849 PMC2604864

[8] McConnell A , “Robotic devices and brain-machine interfaces for hand rehabilitation post-stroke,” J. Rehabil. Med, vol. 49, no. 6, pp. 449–460, Jun. 2017, doi: 10.2340/16501977-2229. 28597018

[9] Lo HS and Xie SQ, “Exoskeleton robots for upper-limb rehabilitation: State of the art and future prospects,” Med. Eng. Phys, vol. 34, no. 3, pp. 261–268, Apr. 2012, doi: 10.1016/j.medengphy.2011.10.004. 22051085

[10] Proietti T, Crocher V, Roby-Brami A, and Jarrassé N, “Upperlimb robotic exoskeletons for neurorehabilitation: A review on control strategies,” IEEE Rev. Biomed. Eng, vol. 9, pp. 4–14, 2016, doi: 10.1109/RBME.2016.2552201. 27071194

[11] Oña ED, Cano-de la Cuerda R, Sánchez-Herrera P, Balaguer C, and Jardón A, “A review of robotics in neurorehabilitation: Towards an automated process for upper limb,” J. Healthcare Eng, vol. 2018, no. 1, Apr. 2018, Art. no. 9758939, doi: 10.1155/2018/9758939. PMC5901488 29707189

[12] Hameed HK, Hassan WZW, Shafie S, Ahmad SA, and Jaafar H, “A review on surface electromyography-controlled hand robotic devices used for rehabilitation and assistance in activities of daily living,” JPO J. Prosthetics Orthotics, vol. 32, no. 1, pp. 3–13, Jan. 2020. [Online]. Available: <Go to ISI>://WOS:000937707000002

[13] Nam C , “An exoneuromusculoskeleton for self-help upper limb rehabilitation after stroke,” Soft Robot, vol. 9, no. 1, pp. 14–35, Feb. 2022, doi: 10.1089/soro.2020.0090. 33271057 PMC8885439

[14] Sukal TM, Ellis MD, and Dewald JPA, “Shoulder abduction-induced reductions in reaching work area following hemiparetic stroke: Neuroscientific implications,” Exp. Brain Res, vol. 183, no. 2, pp. 215–223, Nov. 2007, doi: 10.1007/s00221-007-1029-6. 17634933 PMC2827935

[15] McPherson LM and Dewald JPA, “Differences between flexion and extension synergy-driven coupling at the elbow, wrist, and fingers of individuals with chronic hemiparetic stroke,” Clin. Neurophysiol, vol. 130, no. 4, pp. 454–468, Apr. 2019, doi: 10.1016/j.clinph.2019.01.010. 30771722 PMC7856836

[16] Ellis MD, Schut I, and Dewald JPA, “Flexion synergy overshadows flexor spasticity during reaching in chronic moderate to severe hemiparetic stroke,” Clin. Neurophysiol, vol. 128, no. 7, pp. 1308–1314, Jul. 2017, doi: 10.1016/j.clinph.2017.04.028. 28558314 PMC5507628

[17] Ellis MD, Sukal T, DeMott T, and Dewald JPA, “ACT3D exercise targets gravity-induced discoordination and improves reaching work area in individuals with stroke,” in Proc. IEEE 10th Int. Conf. Rehabil. Robot., Jun. 2007, pp. 890–895, doi: 10.1109/ICORR.2007.4428529.

[18] Masia L, Igo Krebs H, Cappa P, and Hogan N, “Design and characterization of hand module for whole-arm rehabilitation following stroke,” IEEE/ASME Trans. Mechatronics, vol. 12, no. 4, pp. 399–407, Aug. 2007, doi: 10.1109/TMECH.2007.901928. 20228969 PMC2836734

[19] Ellis MD, Lan Y, Yao J, and Dewald JPA, “Robotic quantification of upper extremity loss of independent joint control or flexion synergy in individuals with hemiparetic stroke: A review of paradigms addressing the effects of shoulder abduction loading,” J. NeuroEngineering Rehabil, vol. 13, no. 1, pp. 1–11, Oct. 2016, doi: 10.1186/s12984-016-0203-0. PMC5086410 27794362

[20] Makowski NS, Knutson JS, Chae J, and Crago PE, “Control of robotic assistance using poststroke residual voluntary effort,” IEEE Trans. Neural Syst. Rehabil. Eng, vol. 23, no. 2, pp. 221–231, Mar. 2015, doi: 10.1109/TNSRE.2014.2364273. 25373107

[21] Nasiri R, Aftabi H, and Ahmadabadi MN, “Human-in-the-loop weight compensation in upper limb wearable robots towards total muscles’ effort minimization,” IEEE Robot. Autom. Lett, vol. 7, no. 2, pp. 3273–3278, Apr. 2022, doi: 10.1109/LRA.2022.3144519.

[22] Georgarakis AM, Song J, Wolf P, Riener R, and Xiloyannis M, “Control for gravity compensation in tendon-driven upper limb exosuits,” in Proc. 8th IEEE RAS/EMBS Int. Conf. Biomed. Robot. Biomechtron. (BioRob), Nov. 2020, pp. 340–345. [Online]. Available: <Go to ISI>://WOS:000636920600054

[23] Just F, Özen Ö, Tortora S, Klamroth-Marganska V, Riener R, and Rauter G, “Human arm weight compensation in rehabilitation robotics: Efficacy of three distinct methods,” J. NeuroEng. Rehabil, vol. 17, no. 1, pp. 1–17, Dec. 2020, doi: 10.1186/s12984-020-0644-3. 32024528 PMC7003349

[24] Iandolo R , “Perspectives and challenges in robotic neurorehabilitation,” Appl. Sci, vol. 9, no. 15, p. 3183, Aug. 2019, doi: 10.3390/app9153183.

[25] Washabaugh EP, Treadway E, Gillespie RB, Remy CD, and Krishnan C, “Self-powered robots to reduce motor slacking during upper-extremity rehabilitation: A proof of concept study,” Restorative Neurol. Neurosci, vol. 36, no. 6, pp. 693–708, Nov. 2018, doi: 10.3233/rnn-180830. PMC6817341 30400120

[26] Kopke JV, Ellis MD, and Hargrove LJ, “Feasibility of two different EMG-based pattern recognition control paradigms to control a robot after stroke–case study,” in Proc. 8th IEEE RAS/EMBS Int. Conf. Biomed. Robot. Biomechatron. (BioRob), Nov. 2020, pp. 833–838, doi: 10.1109/BIOROB49111.2020.9224395. PMC8006593 33786207

[27] Scheme E, Lock B, Hargrove L, Hill W, Kuruganti U, and Englehart K, “Motion normalized proportional control for improved pattern recognition-based myoelectric control,” IEEE Trans. Neural Syst. Rehabil. Eng, vol. 22, no. 1, pp. 149–157, Jan. 2014, doi: 10.1109/TNSRE.2013.2247421. 23475378

[28] Hudgins B, Parker P, and Scott RN, “A new strategy for multifunction myoelectric control,” IEEE Trans. Biomed. Eng, vol. 40, no. 1, pp. 82–94, Apr. 1993, doi: 10.1109/10.204774. 8468080

[29] Lan YY, Yao J, and Dewald JPA, “The impact of shoulder abduction loading on EMG-based intention detection of hand opening and closing after stroke,” in Proc. Annu. Int. Conf. IEEE Eng. Med. Biol. Soc., Aug. 2011, pp. 4136–4139. [Online]. Available: <Go to ISI>://WOS:000298810003119 10.1109/IEMBS.2011.6091027 PMC3650847 22255250

[30] Antuvan CW, Ison M, and Artemiadis P, “Embedded human control of robots using myoelectric interfaces,” IEEE Trans. Neural Syst. Rehabil. Eng, vol. 22, no. 4, pp. 820–827, Jul. 2014, doi: 10.1109/TNSRE.2014.2302212. 24760930

[31] Dyson M, Dupan S, Jones H, and Nazarpour K, “Learning, generalization, and scalability of abstract myoelectric control,” IEEE Trans. Neural Syst. Rehabil. Eng, vol. 28, no. 7, pp. 1539–1547, Jul. 2020, doi: 10.1109/TNSRE.2020.3000310. 32634092

[32] Lenzi T, De Rossi SMM, Vitiello N, and Carrozza MC, “Intention-based EMG control for powered exoskeletons,” IEEE Trans. Biomed. Eng, vol. 59, no. 8, pp. 2180–2190, Aug. 2012, doi: 10.1109/TBME.2012.2198821. 22588573

[33] Ellis MD, Sukal-Moulton T, and Dewald JPA, “Progressive shoulder abduction loading is a crucial element of arm rehabilitation in chronic stroke,” Neurorehabil. Neural Repair, vol. 23, no. 8, pp. 862–869, Oct. 2009, doi: 10.1177/1545968309332927. 19454622 PMC2833097

[34] Zhang X , “SEMG-based shoulder-elbow composite motion pattern recognition and control methods for upper limb rehabilitation robot,” Assem. Autom, vol. 39, no. 3, pp. 394–400, Aug. 2019, doi: 10.1108/AA-11-2017-148.

[35] Jiang Y , “Shoulder muscle activation pattern recognition based on sEMG and machine learning algorithms,” Comput. Methods Programs Biomed, vol. 197, Dec. 2020, Art. no. 105721, doi: 10.1016/j.cmpb.2020.105721. 32882593

[36] Rivela D, Scannella A, Pavan EE, Frigo CA, Belluco P, and Gini G, “Analysis and comparison of features and algorithms to classify shoulder movements from sEMG signals,” IEEE Sensors J, vol. 18, no. 9, pp. 3714–3721, May 2018, doi: 10.1109/JSEN.2018.2813434.

[37] Young AJ, Hargrove LJ, and Kuiken TA, “The effects of electrode size and orientation on the sensitivity of myoelectric pattern recognition systems to electrode shift,” IEEE Trans. Biomed. Eng, vol. 58, no. 9, pp. 2537–2544, Sep. 2011, doi: 10.1109/TBME.2011.2159216. 21659017 PMC4234036

[38] Ellis MD, Sukal-Moulton TM, and Dewald JPA, “Impairment-based 3-D robotic intervention improves upper extremity work area in chronic stroke: Targeting abnormal joint torque coupling with progressive shoulder abduction loading,” IEEE Trans. Robot, vol. 25, no. 3, pp. 549–555, Jun. 2009, doi: 10.1109/TRO.2009.2017111. 20657711 PMC2908491

